# Editorial: Advances in Robots Trajectories Learning via Fast Neural Networks

**DOI:** 10.3389/fnbot.2021.671519

**Published:** 2021-03-22

**Authors:** Jose de Jesus Rubio, Yongping Pan, Jeff Pieper, Mu-Yen Chen, Juan Humberto Sossa Azuela

**Affiliations:** ^1^Sección de Estudios de Posgrado e Investigación, ESIME Azcapotzalco, Instituto Politécnico Nacional, Ciudad de Mexico, Mexico; ^2^Department of Biomedical Engineering, National University of Singapore, Singapore, Singapore; ^3^Department of Mechanical and Manufacturing Engineering, University of Calgary, Calgary, AB, Canada; ^4^Department of Engineering Science, National Cheng Kung University, Tainan, Taiwan; ^5^Laboratorio de Robótica y Mecatrónica, Centro de Investigación en Computación, Instituto Politécnico Nacional, Ciudad de Mexico, Mexico

**Keywords:** robots, neural networks, control, trajectory, learning

Motion planning, also known as the navigation problem, is a term used in robotics to find a sequence of valid configurations that moves the robot from the source to a destination. From Figure 1, a robot trajectory may be specified as a sequence of discrete points of a temporal sequence. For this Research Topic, we hope to see focused manuscripts that use artificial intelligence for robots to learn how to develop a trajectory specifically using fast neural networks. Fast neural networks are algorithms which accelerate trajectory learning in robots. Some examples of fast neural networks, and their variants, are long short-term memory, convolutional neural network, recurrent neural network, deep deterministic policy gradient, cascade neural network, genetic algorithm, machine learning, and fuzzy model. The goal of this Research Topic is to welcome research on different types of robots that use fast neural networks to learn and retrieve trajectories in order to perform a positioning task of a robot and contribute solutions to the navigation problems. This Research Topic collected twelve high quality papers reporting the performance results related to some of the previously mentioned emerging research directions how to realize fast neural networks for robots trajectory learning.

**Figure 1:**
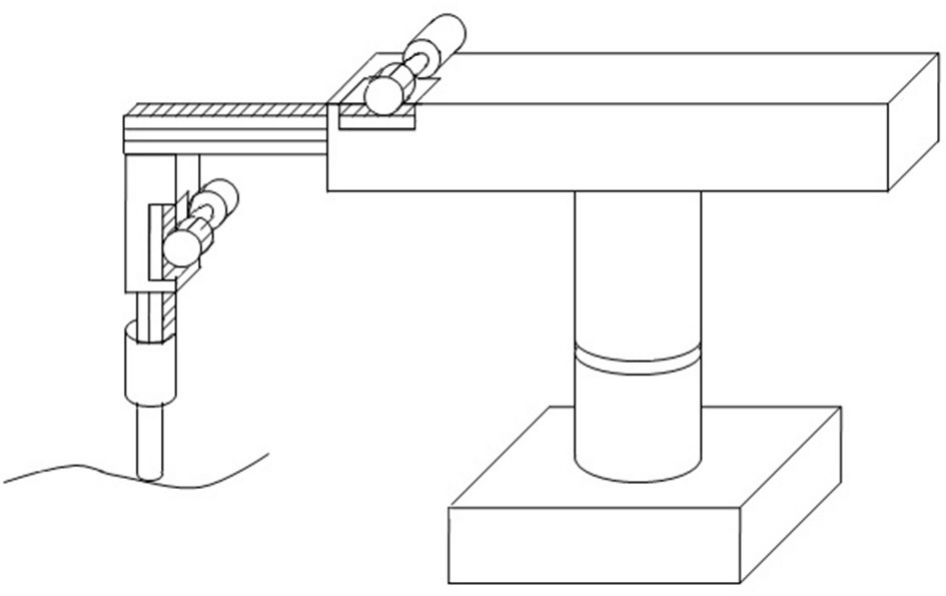
A robot trajectory.

The paper titled “Using Long Short-Term Memory for Building Outdoor Agricultural Machinery” by Wu et al. describes an outdoor agricultural robot that uses Long Short-Term Memory (LSTM). The key features of this innovation consider that the robot is portable and uses green power to reduce installation cost, the system combines the current environment with weather forecasts through LSTM to predict the correct timing for watering, and the robot is mainly for outdoor applications.

The paper titled “Quantum-Based Creative Generation Method for a Dancing Robot” by Mei et al. introduces a creative generation process model based on the quantum modeling simulation method. This model is mainly aimed at generating the running trajectory of a dancing robot and the execution plan of the dancing action. They use digital twin technology to establish the robot trajectory and the dance movements, they use regions with convolutional neural networks to extract character bones and movement features to form a movement library, and the system then render scenes that match the actions through generative adversarial networks.

The paper titled “Bi-criteria Acceleration Level Obstacle Avoidance of Redundant Manipulator” by Zhao et al. presents a recurrent neural network based neural dynamic solver for the improved obstacle-avoidance-scheme-based kinematic control problem in acceleration level for a redundant robot manipulator. The distance between the manipulator and an obstacle is described as the point-to-point distance, and the collision avoidance strategy is formulated as an inequality. From the perspective of optimization, therefore, an acceleration level quadratic programming (QP) problem is formulated to solve the resultant QP minimization problem.

The paper titled “The Path Planning of Mobile Robot by Neural Networks and Hierarchical Reinforcement Learning” by Yu et al. proposes a Deep Deterministic Policy Gradient (DDPG) as a hierarchical reinforcement learning of neural networks for the path planning of mobile robots. Specifically, when compare with Double Deep Q-Learning (DDQN), DDPG has a shorter path planning time and a reduced number of path steps.

The paper titled “PD Control Compensation Based on a Cascade Neural Network Applied to a Robot Manipulator” by Soriano et al. introduces a nominal control law to achieve a sub-optimal performance, and implements a scheme based on a cascade neural network to act as a non-linear compensation of a two-degree-of-freedom robot manipulator. The main contributions of this work are neural compensation based on a cascade neural networks and the function to update the weights.

The paper titled “A Custom EOG-Based HMI Using Neural Network Modeling to Real-Time for the Trajectory Tracking of a Manipulator Robot” by Reynoso et al. considers the generation of points in a Cartesian space (X, Y, Z) in order to control a manipulator robot that follows a desired trajectory by means of the movement of the user eyeball. For this purpose, a multilayer neural network (MNN) is used to model the EOG signal as a mathematical function, which is optimized using genetic algorithms, and the machine learning is customized for the classification in order to reduce the domain time of the system without the need of a database.

The paper titled “CNN Based Detectors on Planetary Environments: A Performance Evaluation” by Furlán et al. presents a convolutional neural network algorithm and an exploration robot for the detection of rocks in environments similar to Mars. The methodology proposed here is based on the use of a Single-Shot-Detector (SSD) network architecture, which has been modified to detect rocks in planetary images.

The paper titled “Optimal UAV's Deployment and Transmit Power Design for Two Users Uplink NOMA Systems” by Zhao describes a simplified setup with two ground users to draw some insightful results in the UAV deployment location. Authors formulate an optimization problem employing the Karush-Kuhn-Tucher (KKT) conditions that maximizes the sum throughput subject to each user transmit power constraint.

The paper titled “Adoption of Machine Learning Algorithm-Based Intelligent Basketball Training Robot in Athlete Injury Prevention” by Xu and Tang proposes a machine learning-based improved Q-Learning algorithm for the path planning obstacle avoidance in an intelligent robot. First, combined with the basketball motion trajectory model, the sport recognition in basketball training was analyzed. Second, the mathematical model of the basketball motion trajectory of the shooting motion is established, and the factors affecting the shooting are analyzed.

The paper titled “Intelligent Badminton Training Robot in Athlete Injury Prevention based on Machine Learning” by Xie et al. presents a machine learning algorithm to explore the role of intelligent badminton training robot (IBTR) in the prevention of badminton player injuries. An IBTR is designed from the perspectives of hardware and software systems, and the movements of the athletes are recognized and analyzed with the hidden Markov model (HMM) in the machine learning.

The paper titled “The Industrial Robot based on Machine Vision and Artificial Intelligence in 5G Environment” by Jin et al. introduces a visual recognition system of industrial robots based on improved Fast R-CNN target detection model. The image layers are convolved and pooled through the deep learning model of artificial intelligence.

To end up, the paper titled “The Analysis of Trajectory Control of Nonholonomic Mobile Robots based on Internet of Things Target Image Enhancement Technology and Backpropagation Neural Network” by Zhao proposes the trajectory tracking and control of incomplete mobile robots. First, the mathematical kinematics model of the nonholonomic mobile robot is studied. Then, the improved Backpropagation Neural Network (BPNN) based on combining the fuzzy algorithm and the neural network is applied to the robot controller.

We conclude this editorial by expressing our sincere gratitude and appreciation to the Specialty Chief Editors of Frontier in Neurorobotics, Alois C. Knoll and Florian Röhrbein, for their great support throughout the compilation of this Research Topic, for their guidance when proposing this Research Topic. Last but not least, we thank the authors for their contributions to this Research Topic and to all reviewers for their voluntary contribution in the peer-review process to maintain a high standard of our Research Topic.

## Author Contributions

JR, YP, and JP: writing—original draft. M-YC and JS: review and editing. All authors have read and agreed to the published version of the manuscript.

## Conflict of Interest

The authors declare that the research was conducted in the absence of any commercial or financial relationships that could be construed as a potential conflict of interest.

